# Genotype‐by‐environment interactions for precopulatory mate guarding in a lek‐mating insect

**DOI:** 10.1002/ece3.6841

**Published:** 2020-09-30

**Authors:** Nikolas Vellnow, Sonja Schindler, Tim Schmoll

**Affiliations:** ^1^ Evolutionary Biology Bielefeld University Bielefeld Germany

**Keywords:** *Achroia grisella*, behavioral plasticity, G × E, inbred lines, lek, mating latency, precopulatory sexual selection, wax moth

## Abstract

In sexually reproducing species, males often experience strong pre‐ and postcopulatory sexual selection leading to a wide variety of male adaptations. One example is mate guarding, where males prevent females from mating with other males either before or after they (will) have mated themselves. In case social conditions vary short term and in an unpredictable manner and if there is genetic variation in plasticity of mate guarding (i.e., genotype‐by‐environment interaction, G × E), adaptive behavioral plasticity in mate guarding may evolve.

Here, we test for genetic variation in the plasticity of precopulatory mate‐guarding behavior in the lek‐mating lesser wax moth *Achroia grisella*. When offered two females in rapid succession, virgin males of this species usually copulate around 10–20 min with the first female. With the second female, however, they engage in copulation posture for many hours until they have produced another spermatophore, an unusual behavior among insects possibly functioning as precopulatory mate guarding. Previous studies showed the mating latency with the second female to be shorter under higher perceived sperm competition risk. We accordingly measured the mate‐guarding behavior of males from six inbred lines under either elevated perceived male–male competition risk or under control conditions allowing us to test for G × E interactions.

We found significant inbred line‐by‐competitor treatment interactions on mating latency and copulation duration with the second female suggesting genetic variation in the degree of behavioral plasticity. However, we found no significant G × E interaction on the sum of mating latency and copulation duration. Our results suggest a potential for adaptive evolution of mate‐guarding plasticity in natural populations of lek‐mating species. Future studies using selection experiments and experimental evolution approaches in laboratory populations, or comparisons of multiple natural populations will be helpful to study under which conditions plasticity in male mate‐guarding behavior evolves.

## Graphical abstract

Sexual selection in lek‐mating species, in which females choose their mates among aggregating males, that simultaneously display their sexual ornaments, is a reasonably well‐studied field. However, only little is known about alternative male mating strategies besides the obvious ornament presentation. In the submitted study, we used the lek‐mating lesser wax moth *Achroia grisella* to study the evolutionary potential of an unusual precopulatory male mate‐guarding behavior. We found significant genotype‐by‐social environment interactions for this precopulatory mate‐guarding behavior, which suggests an evolutionary potential for the plasticity of this behavior.

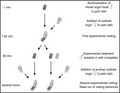

## 1 INTRODUCTION

In sexually reproducing animal species, males often face strong intrasexual selection caused by the competition with other males for access to female mating partners (Andersson, 1994; Darwin, 1871). Since polyandry is the rule rather than the exception in most animal mating systems (Taylor et al., 2014), males often also face strong sperm competition, that is, their ejaculates will continue to compete after copulation for access to unfertilized eggs (Parker, 1970). Sperm competition as an almost ubiquitous evolutionary force has led to the evolution of a wide variety of adaptations in male physiology, ejaculate traits, and behavior (reviewed in Birkhead et al., 2008; Birkhead & Møller, 1998).

One such male adaptation to sperm competition is mate guarding by which males try to prevent females from copulating with other males (Parker, 1970). Males can either exhibit precopulatory or postcopulatory mate guarding. Postcopulatory mate guarding is widespread and takes place subsequent to insemination and prevents sperm competition with sperm from potential rival males the female could mate with in the future (Alcock, 1994; Elias et al., 2014; Parker, 1970). Precopulatory mate guarding on the other hand is relatively rare and consists of the male being closely associated with the female in the period before insemination preventing rival males from access to the female before the focal male has transferred an ejaculate (Jormalainen, 1998; Ridley, 1983). Precopulatory mate guarding is thought to evolve mostly in species in which female encounter rate is low or unpredictable, and female receptivity is restricted to a short and predictable time period (Grafen & Ridley, 1983; Parker, 1970; Ridley, 1983), or in species with first male sperm precedence (Parker, 1974). However, males may also guard females because of reproductive constraints, for example, during an obligatory sexual refractory period between two successive copulations (Greenfield & Coffelt, 1983; Jarrige et al., 2016; Parker & Vahed, 2010). Whatever the specific fitness benefit for males for engaging in precopulatory mate‐guarding behavior may be, it must outweigh any costs to evolve adaptively. Here, opportunity costs due to missed alternative mating opportunities with higher‐quality females may be particularly relevant.

In lek‐mating species, females visit aggregations of males, which simultaneously display their sexual ornaments, and females use these male traits to choose their mates (Darwin, 1871; Höglund & Alatalo, 1995; Kirkpatrick & Ryan, 1991). By exerting often strong directional intersexual selection, females typically create a large variance in male reproductive success and thereby select for exaggerated male sexual signals (Darwin, 1871; Höglund & Alatalo, 1995; Kirkpatrick & Ryan, 1991). In lek‐mating systems, males normally provide females with nothing but sperm (i.e., with no non‐genetic benefits) and males with high mating success may face the risk of sperm depletion. These males may benefit from performing precopulatory mate guarding until they have replenished their sperm supplies and can provide also their next partner with sufficient sperm (Jarrige et al., 2016). Selection pressure to secure available females also under sperm limitation may be high because the operational sex ratio (OSR), that is, the ratio between sexually receptive males and females, is often strongly male‐biased in lek‐mating systems (Emlen & Oring, 1977). However, the operational sex ratio, and therefore the degree of male–male competition, may not be constant throughout the entire breeding season. In fact, short‐term temporal and spatial variation in the operational sex ratio has been shown in natural populations (Carroll, 1993; Kasumovic et al., 2008). It is therefore likely that males in lek‐mating species experience varying degrees of sexual competition risk, for example, depending on the time of day, or stochastic local recruitment of males and females to the lek. Such short‐term‐reversible variation in competition risk may select for short‐term‐reversible *behavioral plasticity* (Carter et al., 2015; Piersma & Drent, 2003) in mate‐guarding behavior (we prefer the term behavioral plasticity as used by Carter et al. (2015) although it could be viewed as a special case of the phenomenon termed *phenotypic flexibility* used by Piersma and Drent (2003)). If there is also genetic variation for the degree of behavioral plasticity, a plastic mate‐guarding behavior may evolve.

Males of the lek‐mating lesser wax moth *Achroia grisella* (Lepidoptera: Pyralidae) show a remarkable precopulatory mate‐guarding behavior when sequentially offered two females in quick succession: While copulations with the first female typically last around 10–20 min (Cordes et al., 2013; Greenfield & Coffelt, 1983), with the second female males engage in copulation posture for several hours, probably because they need longer periods of time to produce the next spermatophore (Greenfield & Coffelt, 1983; Jarrige et al., 2016). To the best of our knowledge, *A. grisella* is the only lek‐mating species in which such a precopulatory mate guarding has been observed. So far, precopulatory mate guarding has been observed mostly in crustaceans, arthropods, where males find immature females and guard them until maturation against competitor males (Elias et al., 2014; Jormalainen, 1998; Potter et al., 1976). In *A. grisella,* the latency to engage in the second copulation has been shown to be shorter and the copulation duration to be longer under higher perceived male–male competition risk (Jarrige et al., 2016). The observed sensitivity to male competitors implies costs of engaging quickly into a second copulation, for example, costs from missed alternative mating opportunities (Jarrige et al., 2016), since in the absence of such costs all males would be predicted to indiscriminately engage in a second copulation without delay.

Unpredictable, short‐term temporal variation in operational sex ratio, which cannot adaptively be responded to with physiological or morphological changes, may select for short‐term behavioral plasticity. However, although strong evidence for the presence of such plasticity in male mating strategies across many taxa exists (Bretman et al., 2011), we are aware of only few studies testing for genetic variation for plasticity in mate‐guarding behavior. We here test for genetic variation in the plasticity of precopulatory mate‐guarding behavior in response to elevated perceived male–male competition risk in the lesser wax moth *A. grisella*. In general, we expect males to increase their mate guarding in the presence of a competitor (Jarrige et al., 2016), although here we studied the presence of genetic variation for this plastic response to the presence of a competitor. We adopted an established experimental paradigm where we sequentially offered a focal male, that belonged to one of six inbred lines, and two virgin outbred females in immediate succession. These two matings were separated by a 30‐min treatment period during which the focal male was either isolated or in close proximity to a male competitor. Subsequently, we measured the mate‐guarding behavior during the mating trial with the second female to test whether males plastically modify their mate‐guarding behavior in response to the presence of a male competitor and whether this plasticity varies between inbred lines.

## 2 MATERIAL AND METHODS

### 2.1 Study species and culture lines

The lesser wax moth *A. grisella* is a small (about 13 mm long), cosmopolitan insect that infests honey bee colonies (Kunike, 1930). Wax moth larvae eat bee larva, pupae, and pollen, but adults have atrophied mouthparts leaving them unable to eat and drink during their short adult life span of 6–18 days for females (TS, unpublished results) and of 10–14 days for males (Greenfield & Coffelt, 1983). Therefore, the somatic and reproductive function of adult moths has to be maintained with a fixed and finite amount of resources (Greenfield & Coffelt, 1983). Males gather in small leks in the vicinity of a hive, for example, on the vertical sides of beehives or on trees adjacent to it, and spend most of their adult life generating costly ultrasound songs to attract females (Greenfield & Coffelt, 1983; Jang & Greenfield, 1998; Reinhold et al., 1998). Females are attracted to the male courtship songs from up to 1–2 meters (Spangler et al., 1984), approach the leks and choose males with certain song trait values, for example, higher amplitude, higher pulse pair rate, and higher asynchrony intervals (Jang & Greenfield, 1996, 1998). Although males also produce pheromones, females are attracted by those pheromones only over long distances and use acoustic cues to choose their mates (Dahm et al., 1971; Spangler et al., 1984). Analyses of female preference thresholds show that the distribution of male song traits lies above female preference thresholds. This suggests that females use a “best‐of‐n” rule to find their mate, that is, they chose the male with the most attractive song traits among the males present at a lek (Brandt et al., 2005). Males often fight with other signaling males, and to the best of our knowledge, there is no relationship between the success during these fights and either attractiveness of males songs or male body mass (Cremer & Greenfield, 1998).

The focal males in our study belonged to six inbred lines, each of which derived from an outbred culture founded with several hundred individuals collected in Tours, Départment Indre et Loire (IL culture), France, in 2007 (Jarrige et al., 2016). Each IL inbred line is the product of 18–20 generations of full‐sib inbreeding, followed by a period when each generation was produced by unconstrained mating between several males and females of the same line (Alem et al., 2013), followed again by a strict full‐sib inbreeding scheme until our experiment began in early 2019. These inbred lines differ significantly in their phenotype, for example, males from different lines differ in their body mass (Figure S1). Male competitors and female mating partners for the focal inbred males belonged to an outbred culture *Indre et Loire New* (ILN culture), which was founded with individuals collected in Tours, Départment Indre et Loire, France, in 2015. The ILN culture is kept in mixed‐age groups in the laboratory since 2015 at room temperature 25°C, humidity 35%, and a photoperiod light:dark 12:12. They are kept in nine rearing boxes in each of which 10–20 adults can mate freely. In order to maintain a high genetic diversity, every 3–4 weeks new boxes are set up with fresh food and a mix of larvae and pupae from three previous boxes, which were not mixed the round before. All moths in this experiment received ad libitum food during their larval stage (following Jarrige et al., 2016) and were kept under a 12:12‐hr light:dark photoperiod.

**FIGURE 1:**
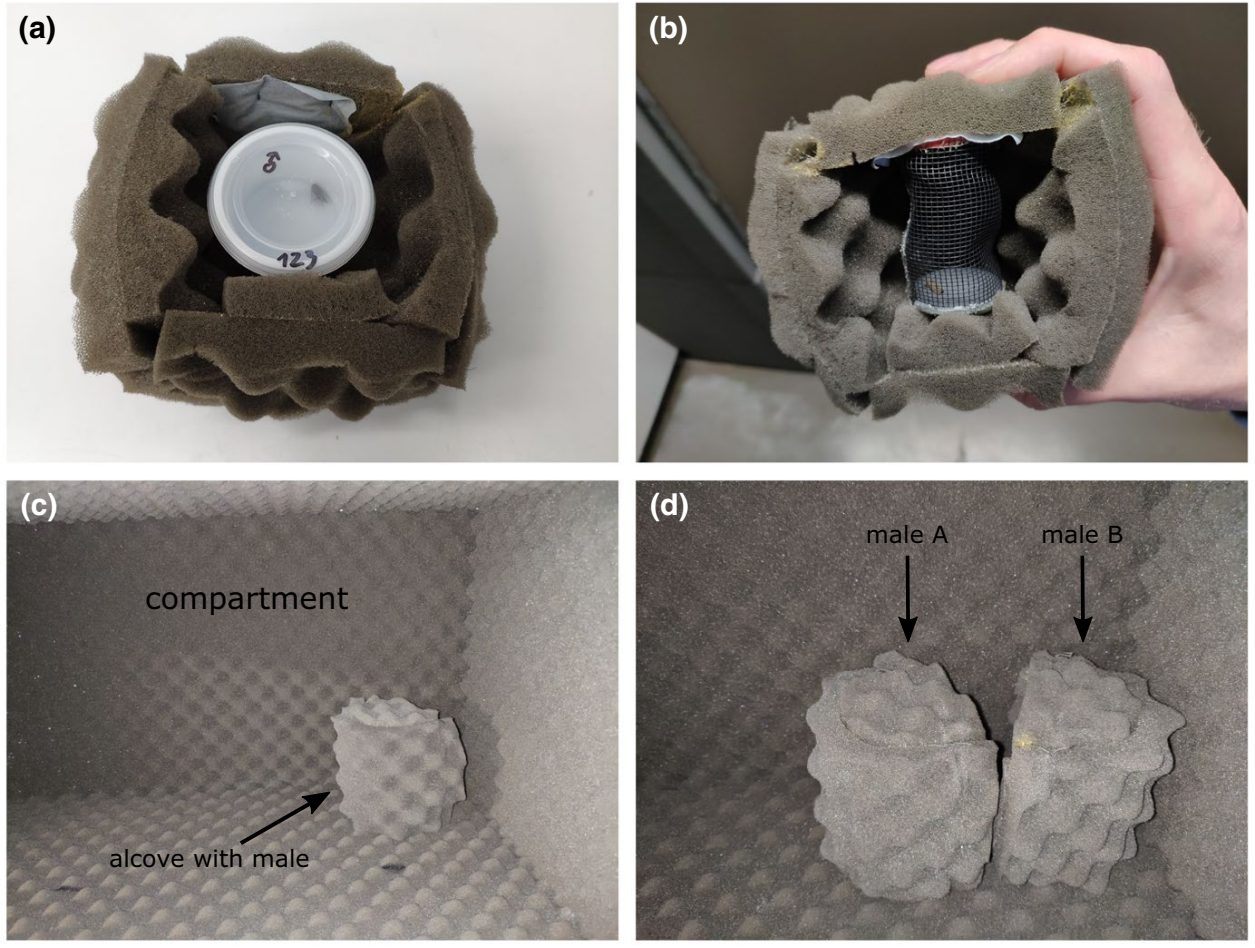
Pictures of the acoustic isolation procedures for focal males before and during the mating trials. (a) We transferred plastic cups including the individuals for the experiment into acoustic foam alcoves as soon as we observed their eclosion. (b) After their first mating, we transferred the males into a small cylindrical net cage, which was placed into an acoustic foam alcove. (c) Males were then placed into a compartment of a shelf lined with acoustic foam. Control males were placed with their alcove directed to a corner of the otherwise empty compartment. (d) Males allocated to the competitor treatment were placed with the opening of the alcove directed to the opening of another alcove containing the competitor male but directed away from the opening of the shelve compartment

To breed focal inbred males, we paired one male and one female from the same IL inbred line (F0) in 28‐mL plastic cups, allowing them to mate and the female to lay eggs. To breed competitor males and female mating partners, we paired one female and one male from different families of the ILN culture and treated them otherwise the same as the F0 generation of the focal inbred males. 21 days after pairing the F0 parents, we transferred hatched larvae (F1) into larger plastic boxes with ad libitum food and allowed them to develop until pupation. We isolated pupae in 28‐mL plastic cups and checked every morning for eclosion. As soon as we observed eclosed moths in the plastic cups, we transferred them (males and females) individually into acoustic foam alcoves to isolate them from sounds of conspecifics (Figure 1a).

### 2.2 Experimental design

**FIGURE 2:**
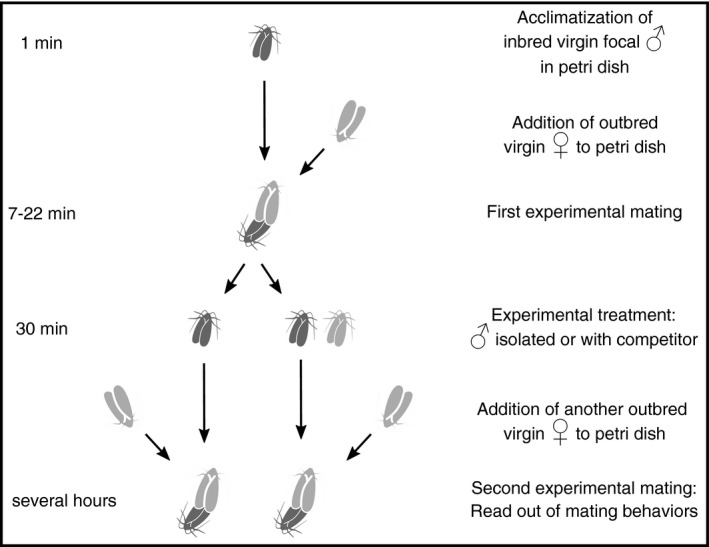
Illustration of the experimental design and experimental procedures. Focal males (dark gray) stemmed from six different Indre et Loire (IL) inbred lines, females (light gray with dotted pattern), and competitor males (light gray) stemmed from an IL outbred culture

All mating trials took place in an acoustically insulated room at 25°C, which has proven itself to be an ideal experimental setting where we can observe similar behaviors as described in the field, starting at the beginning of the scotophase during which most matings occur under natural conditions (Greenfield & Coffelt, 1983). We brought together a focal inbred male with a virgin female of the ILN culture in a clean 60 × 15‐mm glass petri dish (Steriplan, Duran) starting in the first half of their scotophase and allowed them to mate (see Figure 2 for schematic of mating trial design).

Immediately after the pair separated, we took the male out of the petri dish and transferred it into a small cylindrical net cage (30 mm diameter × 55 mm height). We placed the net cages with focal inbred males of the competitor treatment inside an acoustic foam alcove and positioned it next to an outbred competitor, who was also inside a net cage in an acoustic foam alcove (Figure 1d). We positioned both males in a way that they could hear each other's songs but no sounds from other conspecifics (and presumably see each other too). We treated males of the control treatment in the same way but positioned their foam alcoves in a way preventing them from hearing any other moth (Figure 1c). We then removed the first female from the petri dish. After focal inbred males were subjected to either of these treatments for 30 min, we paired them to a second virgin ILN female. We video‐taped this second mating trial in the same petri dish until the mid of their next photophase with one frame per second using an action camera attached to a tripod (SJCAM Sj4000 and Cullmann NANOMAX 400T, respectively).

We started mating trials for a total of 35 focal inbred males from the different IL lines but excluded three: one because the copulation in the first mating trial lasted unusually long (over seven hours), one because the male was unable to mate during the first mating trial due to its small body size, and one because the male escaped after the first mating trial. The final sample sizes for the respective inbred lines were as follows: IL11 = 5, IL115 = 9, IL129 = 4, IL3 = 4, IL37 = 3, and IL44 = 7.

By analyzing the videos of the second mating trial, we estimated the time from placing the male and female together until they started copulating (*mating latency* hereafter), the time they remained *in copula* (*copulation duration* hereafter), and the sum of the mating latency and the copulation duration (*total time* hereafter) to the nearest second. We consider the copulation duration under this experimental design as a measure of male mate guarding sensu stricto with males that copulate for longer exhibiting a higher degree of mate guarding. Mating latency on the other hand also belongs to the general mate‐guarding behavior and can be considered mate guarding sensu lato with males with short mating latencies exhibiting a high degree of mate guarding vice versa. Males usually copulate immediately after mounting and did so in this experiment (N.V. pers. obs.). We consider mating latency to be mainly under male control in our experiment because females were virgin and therefore very eager to mate (N.V. pers. obs.). Generally, only already mated females (which we did not use in this study) may reject male mating attempts (Engqvist et al., 2014). The person analyzing the videos (N.V.) was blind with respect to the inbred line of the focal male and the treatment it had experienced.

### 2.3 Statistical analysis

We used R 3.6.1 (R Development Core Team, 2019) to fit linear models with either mating latency, copulation duration, or total time during the second mating trial as response variables. To improve model fit we log‐transformed mating latency (adding a constant of one second to all values), which had a heavily right‐skewed distribution. In order to test for genetic variation in how males adjust their mate‐guarding behavior to sexual competition risk, we fitted three linear models for each response variable: (a) a null model including only an intercept, (b) a model with only the main effects of treatment and inbred line, and (c) the full model with the main effects and the treatment × inbred line interaction. First, we compared both the main effects model and the full model with the null model for each response variable using likelihood‐ratio tests (Zeileis & Hothorn, 2002). Then, we compared the full model with the main effects model. Comparing the full model with both the null model and the main effects, model gave qualitatively very similar results (Table 1). Since our main hypothesis is tested by the interaction effect between treatment and inbred line, and because interpretation of main effects in the presence of significant interactions is considered difficult, we focus on the comparison between full and main effects models in our results and discussion (Engqvist, 2005; Gelman & Hill, 2006; Schielzeth, 2010; Sokal & Rohlf, 1995).

**TABLE 1:** Comparisons between linear models with either (i) the null model including only an intercept term, (ii) the additive main effects of treatment and inbred line or with (iii) the main effects and their interaction for mating latency, copulation duration, and their sum (total time) in second experimental matings

Trait	Model	Model *df*	LogLik	Comparison to null model			Comparison to main effects model		
				*df*	*χ* ^2^	*p*‐value	*df*	*χ* ^2^	*p*‐value
Mating latency	Intercept	2	−70.7	—	—	—	—	—	—
	Treatment + inbred line	8	−66.8	6	7.82	0.25	—	—	—
	Treatment × inbred line	13	−57.1	11	27.2	0.0042	5	19.4	0.0016
Copulation duration	Intercept	2	−193.8	—	—	—	—	—	—
	Treatment + inbred line	8	−190.3	6	7.05	0.32	—	—	—
	Treatment × inbred line	13	−184.2	11	19.4	0.054	5	12.3	0.030
Total time	Intercept	2	−193.5	—	—	—	—	—	—
	Treatment + inbred line	8	−188.6	6	9.78	0.13	—	—	—
	Treatment × inbred line	13	−183.6	11	19.7	0.050	5	9.90	0.078

Note

Likelihood‐ratio tests were used for model comparisons (*n* = 32 males from six inbred lines for all traits).

## 3 RESULTS

**FIGURE 3:**
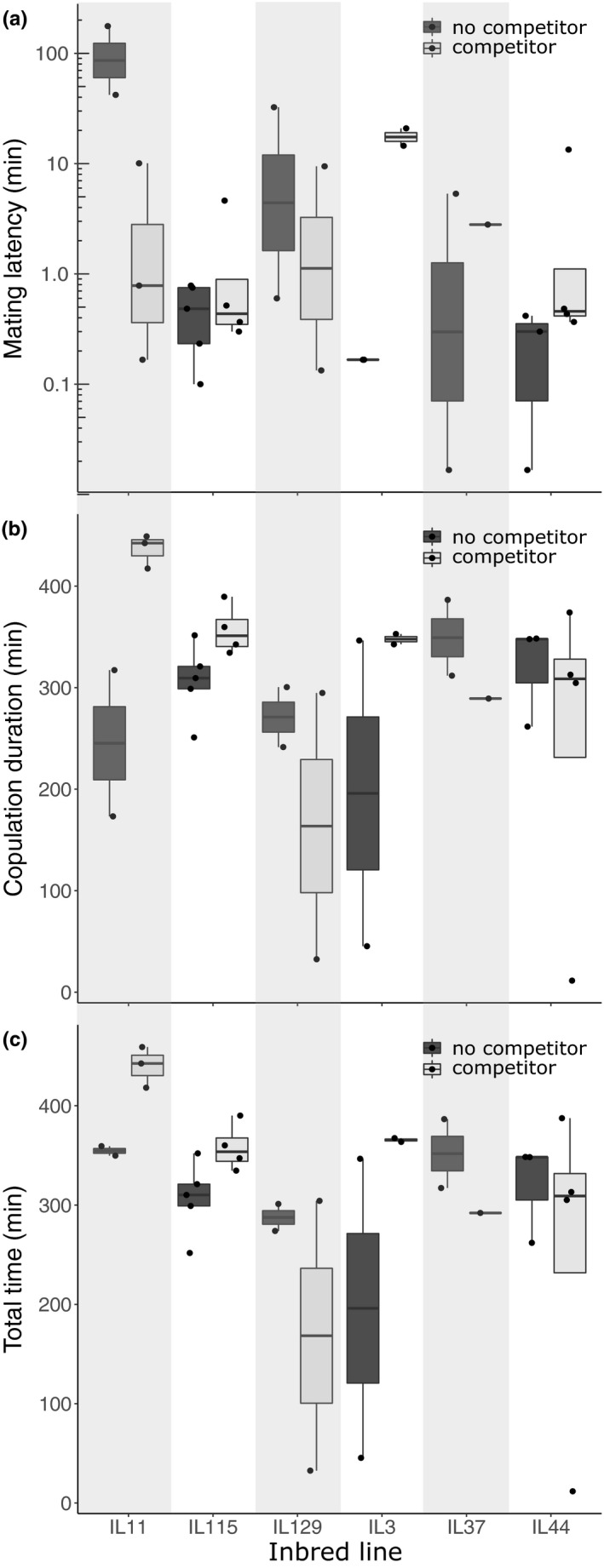
Mating behaviors during the second experimental mating for the six Indre et Loire (IL) inbred lines. Focal males experienced an outbred, virgin male competitor (light gray), or no competitor (dark gray) for 30 min subsequent to terminating their first experimental mating. Shown are the results for (a) mating latency, (b) copulation duration, and (c) their sum (total time). Note that the *y*‐axis in (a) is on a logarithmic scale in order to visualize both very short and very long mating latencies

Overall, mating latencies during second mating trials of males from inbred lines ranged from 1 s to 176 min with a median of 29 s. Males from different inbred lines reacted differently to the presence of a competitor as evident from a significant treatment × inbred line interaction effect on mating latency **(** *χ* ^2^ = 19.4, *df* = 5, *p* = .002, Table 1, Figure 3a).

Overall, copulation durations in second mating trials ranged from 11 to 449 min with a median of 319 min. Copulation duration was also significantly affected by the treatment × inbred line interaction (*χ* ^2^ = 12.3, *df* = 5, *p* = .030, Table 1, Figure 3b).

Overall, the total time from beginning of the second mating trial until the end of the copulation ranged from 12 to 459 min with a median of 341 min overall. The treatment × inbred line interaction on total time was not significant (*χ* ^2^ = 9.90, *df* = 5, *p* = .078, Table 1, Figure 3c).

Mating latency did not correlate with copulation duration (Kendal's *τ *= −0.04; *p* = .77) or with total time (Kendal's *τ *= 0.11, *p* = .36). Copulation duration, however, did correlate strongly with total time (Pearson's *r *= 0.95, *p* < .0001).

## 4 DISCUSSION

We found a significant inbred line‐by‐male competitor treatment interaction on mating latency in the second mating. Especially, males of the IL11 genotype seem to substantially reduce their otherwise relatively long mating latency when they had the opportunity to sense a competitor (cf. Figure 3), while males of some other inbred lines seem to mostly lack the sensitivity to the presence of male competition. Instead, they showed very short mating latencies independent of the presence of a competitor. This difference between the plasticity of IL11 and the other lines seems to be a very large effect. Furthermore, we also found a significant inbred line‐by‐male competitor treatment interaction on copulation duration, most likely as a direct consequence of the effect on mating latency as both variables in sum (i.e., total time) presumably reflect the time required to produce another spermatophore (Greenfield & Coffelt, 1983). The inbred line‐by‐male competitor treatment interaction on mating latency is statistically highly significant. We thus think that our results suggest the possibility of interesting new research approaches to study the evolution of behavioral plasticity and that they corroborate the interpretation of results regarding this mating behavior in earlier studies of the lesser wax moth. In the following, we discuss both topics in turn.

### 4.1 Prolonged copulation duration as precopulatory mate guarding

The fact that there was no significant G × E effect on total time (Table 1, Figure 3c) corroborates the interpretation of the observed extra‐ordinarily long second copulation periods as precopulatory mate‐guarding behavior (Greenfield & Coffelt, 1983; Jarrige et al., 2016): It may take *A. grisella* males several hours after their first mating to produce the next spermatophore and they apparently cannot plastically increase the speed of spermatophore production in response to a competitor. Consequently, they show no strong genetic variation in the plasticity of their total time. However, they can start copulating earlier when they perceive a competitor and then simply stay *in copula* until they can finally transfer the next spermatophore, thereby guarding their mate against competitors before/during sperm transfer. Therefore, we showed that male lesser wax moths, at least from the sampled population at Indre et Loire in Tours, vary genetically in the plasticity of their precopulatory mate‐guarding response to competitors. In particular, they seem to vary genetically in the plasticity of their *behavioral* response, that is, in their mating latency and copulation duration. Whether or not they exhibit genetic variation in the plasticity of their spermatophore production duration, which may be indicated by their total time, is less clear. We think that total time may indicate spermatophore production duration because (a) males show such a long total time during their second mating, but when forcibly separated 4, 5 hr 30 min, or 7 hr after the copulation start, no transferred spermatophores have been found in the females (Greenfield & Coffelt, 1983) and (b) males respond to socio‐ecological context by adjusting mating latency and copulation duration but not total time (Jarrige et al., 2016). We did not find any evidence for a negative correlation between mating latency and copulation duration, which one might expect under the above‐described hypotheses. However, a strong among‐line variation in total time, as present in our study, might mask such a negative correlation.

### 4.2 Evolution of plasticity in male mating behavior

The presence of standing genetic variation for behavioral plasticity indicates that natural populations of the lesser wax moth may show an evolutionary response to selection on this behavioral plasticity. If, for example, the OSR (Emlen & Oring, 1977) fluctuates within an individual's lifetime, males may evolve to adjust their mate‐guarding behavior in a plastic manner depending on the level of sexual competition. If, however, the operational sex ratio is constantly either low or high over time, plasticity in male mate‐guarding behavior may not evolve in the first place. And if the maintenance of plasticity is costly, males will be selected to be inflexible and exhibit either constantly low or high levels of mate guarding, respectively. The OSR in natural populations can vary strongly throughout the breeding season and among sampling sites (Carroll, 1993; Kasumovic et al., 2008). This variation has also been observed in lek‐mating species, such as the tarantula hawk wasps (Alcock, 1981), razorbills (Wagner, 1992), and the natterjack toad (Tejedo, 1988). Therefore, the evolution of plastic mate‐guarding behavior seems to be a likely outcome in many natural populations. Unfortunately, we know little about the variation in OSR in natural populations of *A. grisella*. Although Greenfield and Coffelt (1983) suggest a male‐biased sex ratio at the lek they do not report how the OSR varies. We are aware of no study that reported on variation in OSR in *A. grisella* under natural conditions. However, the presence of genetic variation in mate‐guarding plasticity in the present study opens up the opportunity for interesting future research, for example, artificial selection or experimental evolution studies in the laboratory. Here, replicated *A. grisella* populations that evolve under either constantly low or high OSR conditions could be compared with replicated populations that evolve under conditions in which the OSR varies unpredictably between low and high values.

Besides our study, we are aware of only a few studies examining G × E effects on male mating behaviors. There was genetic variation in male reproductive tactics in the burying beetle *Nicrophorus vespilloides*, with interactions between selection line and male competitive environment affecting on‐carcass activity and signaling behavior (Carter et al., 2015). Bretman et al. (2014) failed, however, to find significant interaction effects between isolines and the presence of a male competitor on copulation duration in a well‐replicated study in *Drosophila pseudoobscura*. Similarly, Taylor et al. (2013) found neither significant effects of genotype nor interaction effects between genotype and the social environment (i.e., the presence or absence of a rival male during adult sexual maturation) on male mating behaviors in a well‐replicated quantitative genetic study on *D. melanogaster*. Taken together, this paucity of empirical studies on G × E effects on male mating behavior is unfortunate since the presence of such genetic variation determines whether a given population can evolve a higher behavioral plasticity in response to strong temporal variation in the strength of male–male competition. We are aware of no study besides our testing for G × E effects on male mate‐guarding behavior. However, a study about the soapberry bug, *Jadera haematoloma*, in which males perform a postcopulatory mate guarding by prolonging copulation after sperm transfer, provides interesting evidence for how plasticity in male mate‐guarding behavior may evolve. In a common garden experiment, males from a population with a more variable OSR exhibited more plasticity in their mate‐guarding behavior than males of another population (Carroll & Corneli, 1995). This corroborates the hypothesis that an environment in which the OSR varies unpredictably and on a short time scale selects for a more plastic male mate‐guarding response. Unfortunately, comparing only two natural populations leaves alternative explanation for the difference between these two populations. Studying several, independent populations or even creating independently evolving populations using an experimental evolution approach would provide better evidence for how unpredictable, short‐term variation in the OSR affects the evolution of plasticity in mate‐guarding behavior.

Differences between genotypes in the behavioral plasticity in response to male competition could have evolved as alternative strategies (Gross, 1996). For instance, genotypes that code for male phenotypes that are unattractive to females may optimize their fitness by increasing mate guarding in response to male competition. This is because they are unlikely to be chosen by the female and to find another female willing to mate with them. More attractive male genotypes can possibly be less responsive to male competition because they have a high probability of finding another female that will mate with them. Since male mass in *A. grisella* is positively correlated with attractiveness (Jang & Greenfield, 1998), one might expect that lighter males exhibit more plasticity in their mate‐guarding behavior than heavy males. This is indeed what we find as the lines IL11 and IL115 are the lightest and show the strongest degree of plasticity (cf. Figure 3 & S1). It would be interesting to study this in future experiments with added measures of attractiveness based on male courtship songs to get a more precise estimate of male attractiveness than male mass alone can give.

### 4.3 Conclusions

Males in lek‐mating species may evolve plastic mate‐guarding behavior in response to an unpredictably varying degree of male–male competition. However, there is still only little empirical research about how and under what conditions plasticity in male mating behavior in general, and in male mate guarding in particular, evolves. Studying multiple, independent populations that vary in OSR (or sperm competition risk) and using experimental evolution may provide crucial insights to understand the evolution of plastic male mate‐guarding behavior.

## CONFLICT OF INTEREST

The authors declare to have no conflict of interests.

## AUTHOR CONTRIBUTIONS

**Nikolas Vellnow:** Conceptualization (lead); data curation (lead); formal analysis (lead); funding acquisition (equal); investigation (lead); methodology (equal); resources (equal); software (lead); supervision (equal); validation (lead); visualization (lead); writing – original draft (lead); writing – review and editing (lead). **Sonja Schindler:** Conceptualization (supporting); methodology (supporting); project administration (supporting); resources (equal); writing – review and editing (supporting). **Tim Schmoll:** Conceptualization (supporting); data curation (supporting); formal analysis (supporting); funding acquisition (equal); investigation (equal); methodology (equal); supervision (equal); validation (supporting); visualization (supporting); writing – review and editing (supporting).

## Supporting information

Figure S1

## Data Availability

The data analyzed for this study will be published on the dryad data depository (https://doi.org/10.5061/dryad.r4xgxd29k).

## References

[ece36841-bib-0001] Alcock, J. (1981). Lek territoriality in the tarantula hawk wasp *Hemipepsis ustulata* (Hymenoptera: Pompilidae). Behavioral Ecology and Sociobiology, 8, 309–317. 10.1007/BF00299531

[ece36841-bib-0002] Alcock, J. (1994). Postinsemination associations between males and females in insects: The mate‐guarding hypothesis. Annual Review of Entomology, 39(1), 1–21. 10.1146/annurev.en.39.010194.000245

[ece36841-bib-0003] Alem, S. , Streiff, R. , Courtois, B. , Zenboudji, S. , Limousin, D. , & Greenfield, M. D. (2013). Genetic architecture of sensory exploitation: QTL mapping of female and male receiver traits in an acoustic moth. Journal of Evolutionary Biology, 26, 2581–2596. 10.1111/jeb.12252 24118224

[ece36841-bib-0004] Andersson, M. B. (1994). Sexual selection. : Princeton University Press.

[ece36841-bib-0005] Birkhead, T. R. , Hosken, D. J. , & Pitnick, S. S. (2008). Sperm biology: An evolutionary perspective. Cambridge, MA: Academic Press.

[ece36841-bib-0006] Birkhead, T. R. , & Møller, A. P. (1998). Sperm competition and sexual selection. London, UK: Academic Press Inc.

[ece36841-bib-0007] Brandt, L. S. E. , Ludwar, B. C. , & Greenfield, M. D. (2005). Co‐occurrence of preference functions and acceptance thresholds in female choice: Mate discrimination in the lesser wax moth. Ethology, 111, 609–625. 10.1111/j.1439-0310.2005.01085.x

[ece36841-bib-0008] Bretman, A. , Gage, M. J. G. , & Chapman, T. (2011). Quick‐change artists: Male plastic behavioural responses to rivals. Trends in Ecology & Evolution, 26, 467–473. 10.1016/j.tree.2011.05.002 21680050

[ece36841-bib-0009] Bretman, A. , Lizé, A. , Walling, C. A. , & Price, T. A. R. (2014). The heritability of mating behaviour in a fly and its plasticity in response to the threat of sperm competition. PLoS One, 9, e90236 10.1371/journal.pone.0090236 24587294 PMC3934992

[ece36841-bib-0010] Carroll, S. P. (1993). Divergence in male mating tactics between two populations of the soapberry bug: I. Guarding versus nonguarding. Behavioral Ecology, 4(2), 156–164. 10.1093/beheco/4.2.156

[ece36841-bib-0011] Carroll, S. P. , & Corneli, P. S. (1995). Divergence in male mating tactics between two populations of the soapberry bug: II. Genetic change and the evolution of a plastic reaction norm in a variable social environment. Behavioral Ecology, 6, 46–56.

[ece36841-bib-0012] Carter, M. J. , Head, M. L. , Moore, A. J. , & Royle, N. J. (2015). Behavioral plasticity and G × E of reproductive tactics in *Nicrophorus vespilloides* burying beetles. Evolution, 69, 969–978. 25654994 10.1111/evo.12619 PMC5024017

[ece36841-bib-0013] Cordes, N. , Yiğit, A. , Engqvist, L. , & Schmoll, T. (2013). Differential sperm expenditure reveals a possible role for post‐copulatory sexual selection in a lekking moth. Ecology and Evolution, 3, 503–511. 10.1002/ece3.458 23531777 PMC3605841

[ece36841-bib-0014] Cremer, S. , & Greenfield, M. D. (1998). Partitioning the components of sexual selection: Attractiveness and agonistic behaviour in male wax moths, *Achroia grisella* (Lepidoptera: Pyralidae). Ethology, 104, 1–9. 10.1111/j.1439-0310.1998.tb00025.x

[ece36841-bib-0015] Dahm, K. H. , Meyer, D. , Finn, W. E. , Reinhold, V. , & Röller, H. (1971). The olfactory and auditory mediated sex attraction in *Achroia grisella* (Fabr.). Naturwissenschaften, 58, 265–266. 10.1007/BF00602990 5580885

[ece36841-bib-0016] Darwin, C. (1871). The descent of man, and selection in relation to sex. John Murray.

[ece36841-bib-0017] Elias, D. O. , Sivalinghem, S. , Mason, A. C. , Andrade, M. C. B. , & Kasumovic, M. M. (2014). Mate‐guarding courtship behaviour: Tactics in a changing world. Animal Behaviour, 97, 25–33. 10.1016/j.anbehav.2014.08.007

[ece36841-bib-0018] Emlen, S. T. , & Oring, L. W. (1977). Ecology, sexual selection, and the evolution of mating systems. Science, 197, 215–223. 10.1126/science.327542 327542

[ece36841-bib-0019] Engqvist, L. (2005). The mistreatment of covariate interaction terms in linear model analyses of behavioural and evolutionary ecology studies. Animal Behaviour, 70, 967–971. 10.1016/j.anbehav.2005.01.016

[ece36841-bib-0020] Engqvist, L. , Cordes, N. , Schwenniger, J. , Bakhtina, S. , & Schmoll, T. (2014). Female remating behavior in a lekking moth. Ethology, 120, 662–671. 10.1111/eth.12237

[ece36841-bib-0021] Gelman, A. , & Hill, J. (2006). Data analysis using regression and multilevel/hierarchical models. : Cambridge University Press.

[ece36841-bib-0022] Grafen, A. , & Ridley, M. (1983). A model of mate guarding. Journal of Theoretical Biology, 102, 549–567. 10.1016/0022-5193(83)90390-9

[ece36841-bib-0023] Greenfield, M. D. , & Coffelt, J. A. (1983). Reproductive behaviour of the lesser waxmoth, *Achroia grisella* (Pyralidae: Galleriinae): Signalling, pair formation, male interactions, and mate guarding. Behaviour, 84, 287–315. 10.1163/156853983X00534

[ece36841-bib-0024] Gross, M. R. (1996). Alternative reproductive strategies and tactics: Diversity within sexes. Trends in Ecology & Evolution, 11, 92–98. 10.1016/0169-5347(96)81050-0 21237769

[ece36841-bib-0025] Höglund, J. , & Alatalo, R. V. (1995). Leks. Princeton University Press.

[ece36841-bib-0026] Jang, Y. , & Greenfield, M. D. (1996). Ultrasonic communication and sexual selection in wax moths: Female choice based on energy and asynchrony of male signals. Animal Behaviour, 51, 1095–1106. 10.1006/anbe.1996.0111

[ece36841-bib-0027] Jang, Y. , & Greenfield, M. D. (1998). Absolute versus relative measurements of sexual selection: Assessing the contributions of ultrasonic signal characters to mate attraction in lesser wax moths, *Achroia grisella* (lepidoptera: Pyralidae). Evolution, 52, 1383–1393. 28565373 10.1111/j.1558-5646.1998.tb02020.x

[ece36841-bib-0028] Jarrige, A. , Kassis, A. , Schmoll, T. , & Goubault, M. (2016). Recently mated males of a lek‐mating insect intensify precopulatory mate guarding under male competition. Animal Behaviour, 117, 21–34. 10.1016/j.anbehav.2016.04.012

[ece36841-bib-0029] Jormalainen, V. (1998). Precopulatory mate guarding in crustaceans: Male competitive strategy and intersexual conflict. The Quarterly Review of Biology, 73(3), 275–304. 10.1086/420306

[ece36841-bib-0030] Kasumovic, M. M. , Bruce, M. J. , Andrade, M. C. B. , & Herberstein, M. E. (2008). Spatial and temporal demographic variation drives within‐season fluctuations in sexual selection. Evolution, 62, 2316–2325. 10.1111/j.1558-5646.2008.00446.x 18564373

[ece36841-bib-0031] Kirkpatrick, M. , & Ryan, M. J. (1991). The evolution of mating preferences and the paradox of the lek. Nature, 350(6313), 33–38. 10.1038/350033a0

[ece36841-bib-0032] Kunike, G. (1930). Zur Biologie der kleinen Wachsmotte, *Achroea grisella* Fabr. Zeitschrift Für Angewandte Entomologie, 16, 304–356. 10.1111/j.1439-0418.1930.tb00139.x

[ece36841-bib-0033] Parker, D. J. , & Vahed, K. (2010). The intensity of pre‐ and post‐copulatory mate guarding in relation to spermatophore transfer in the cricket *Gryllus bimaculatus* . Journal of Ethology, 28, 245–249. 10.1007/s10164-009-0176-6

[ece36841-bib-0034] Parker, G. A. (1970). Sperm competition and its evolutionary consequences in the insects. Biological Reviews, 45, 525–567. 10.1111/j.1469-185X.1970.tb01176.x

[ece36841-bib-0035] Parker, G. A. (1974). Courtship Persistence and Female‐Guarding as Male Time Investment Strategies. Behaviour, 48(1‐4), 157–183. 10.1163/156853974X00327

[ece36841-bib-0036] Piersma, T. , & Drent, J. (2003). Phenotypic flexibility and the evolution of organismal design. Trends in Ecology & Evolution, 18, 228–233. 10.1016/S0169-5347(03)00036-3

[ece36841-bib-0037] Potter, D. A. , Wrensch, D. L. , & Johnston, D. E. (1976). Guarding, aggressive behavior, and mating success in male twospotted spider mites. Annals of the Entomological Society of America, 69, 707–711.

[ece36841-bib-0038] R Development Core Team (2019). R: A language and environment for statistical computing. R Foundation for Statistical Computing.

[ece36841-bib-0039] Reinhold, K. , Greenfield, M. D. , Jang, Y. W. , & Broce, A. (1998). Energetic cost of sexual attractiveness: Ultrasonic advertisement in wax moths. Animal Behavior, 55, 905–913. 10.1006/anbe.1997.0594 9632477

[ece36841-bib-0040] Ridley, M. (1983). The explanation of organic diversity: The comparative method and adaptations for mating. Oxford University Press.

[ece36841-bib-0041] Schielzeth, H. (2010). Simple means to improve the interpretability of regression coefficients. Methods in Ecology and Evolution, 1, 103–113. 10.1111/j.2041-210X.2010.00012.x

[ece36841-bib-0042] Sokal, R. R. , & Rohlf, J. F. (1995). Biometry (3rd ed) W. H. Freeman.

[ece36841-bib-0043] Spangler, H. G. , Greenfield, M. D. , & Takessian, A. (1984). Ultrasonic mate calling in the lesser wax moth. Physiological Entomology, 9, 87–95. 10.1111/j.1365-3032.1984.tb00684.x

[ece36841-bib-0044] Taylor, M. L. , Evans, J. P. , & Garcia‐Gonzalez, F. (2013). No evidence for heritability of male mating latency or copulation duration across social environments in *Drosophila melanogaster* . PLoS One, 8, e77347 10.1371/journal.pone.0077347 24155948 PMC3796456

[ece36841-bib-0045] Taylor, M. L. , Price, T. A. R. , & Wedell, N. (2014). Polyandry in nature: A global analysis. Trends in Ecology & Evolution, 29, 376–383. 10.1016/j.tree.2014.04.005 24831458

[ece36841-bib-0046] Tejedo, M. (1988). Fighting for females in the toad *Bufo calamita* is affected by the operational sex ratio. Animal Behaviour, 36, 1765–1769. 10.1016/S0003-3472(88)80115-5

[ece36841-bib-0047] Wagner, R. H. (1992). Extra‐pair copulations in a lek: The secondary mating system of monogamous razorbills. Behavioral Ecology and Sociobiology, 31, 63–71. 10.1007/BF00167817

[ece36841-bib-0048] Zeileis, A. , & Hothorn, T. (2002). Diagnostic checking in regression relationships. R News, 2, 7–10.

