# Supplementary material for: Genotype‐by‐environment interactions for precopulatory mate guarding in a lek‐mating insect
Source: Ecol Evol. 2020 Sep 30;10(21):12138–46. doi: 10.1002/ece3.6841 (PMC7663981; doi:10.1002/ece3.6841)
Supplement: Supplementary file 1 — Figure S1 [file ECE3-10-12138-s001.docx]

Supplementary information for:

GENOTYPE-BY-ENVIRONMENT INTERACTIONS FOR PRECOPULATORY mate guarding in a lek-MATING insect

Male body mass of inbred lines

We measured the body mass of 151 males from the six inbred IL lines to the nearest 0.01 mg with an electronic scale Kern 770 (Kern & Sohn GmbH, Balingen, Germany). To analyze the fata we fitted linear models with inbred line as categorical predictor variable and male body mass as response variable and tested the significance of the inbred line effect by comparing with a likelihood ratio test to the null model.

Males from the six inbred lines differed significantly in their weight (*χ*² = 35.6, d.f. = 5, p < 0.0001, Fig. 1). For instance, males form IL44 were on average 19.3% heavier than males from IL115.


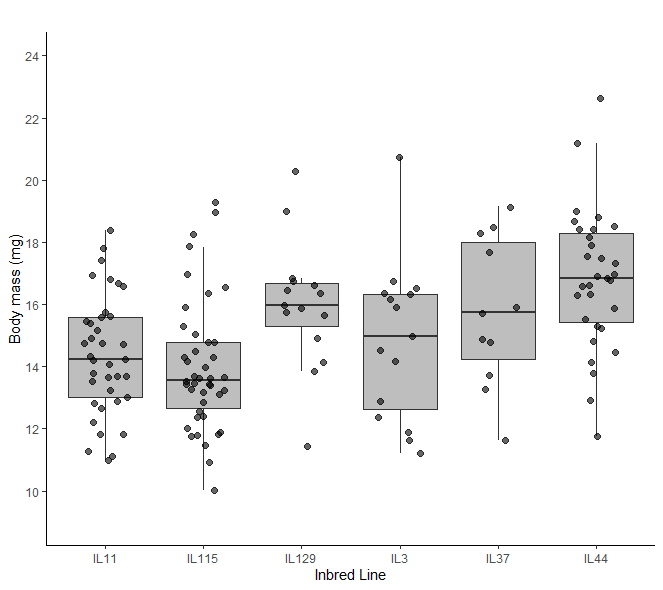


**Figure S1.** Shown are the body mass values from a total of 151 male moths of six inbred lines (IL11: n=37, IL115: n=42, IL129: n=15, IL3: n=15, IL37: n=11, IL44: n=31).
